# Does preoperative patient’s estimated acceptable pain affect the satisfaction with postoperative pain management?

**DOI:** 10.1186/s40981-016-0075-0

**Published:** 2017-01-10

**Authors:** Marie Shigematsu-Locatelli, Takashi Kawano, Sonoe Kitamura, Atsushi Nishigaki, Daiki Yamanaka, Bun Aoyama, Hiroki Tateiwa, Masataka Yokoyama

**Affiliations:** Department of Anesthesiology and Intensive Care Medicine, Kochi Medical School, Kohasu, Oko-cho, Nankoku, Kochi 783-8505 Japan

**Keywords:** Postoperative pain, Numerical rating scale, Patient satisfaction

## Abstract

**Background:**

Patient satisfaction with postoperative pain management is an important quality indicator in patient health care, but its determinants are poorly understood. Here, we examined the contribution of the discrepancy between an individual’s estimated acceptable and actual postoperative pain scores to the overall satisfaction with pain treatment.

**Findings:**

A total of 93 surgical patients were included in this study. Preoperatively, the subjects were asked to rate their estimated acceptable postoperative pain using a numerical rating scale (NRS). One day after the surgery, the patients were again asked to give NRS ratings of the overall actual pain intensity they had experienced, as well as their satisfaction with the provided pain treatment. The median estimated acceptable and actual NRS values for postoperative pain were 4.0 (3.0–5.0) and 4.0 (2.0–5.0), respectively. Although there was no correlation between the degree of patient satisfaction and preoperative estimated acceptable pain intensity, there was a significant negative correlation between the degree of patient satisfaction and postoperative actual pain intensity. When the preoperative estimated acceptable NRS value was compared with the postoperative actual value for each individual, postoperative NRS was greater in 34 cases (36.6%), less in 43 cases (46.2%), and equal in 16 cases (17.2%). The degree of patient satisfaction was not significantly correlated with the magnitude of difference between preoperative estimated acceptable NRS and postoperative actual NRS.

**Conclusions:**

Our findings suggest that inquiring about the estimated acceptable pain before surgery may not help anesthesiologists to understand the patient’s goal of pain management for improving patient satisfaction.

## Findings

### Introduction

Patient satisfaction is widely used as an index to evaluate the quality of medical approaches and hospital services [1, 2]. The outcomes of postoperative pain management have been reported to be a particularly important factor influencing inpatient levels of satisfaction with hospitals [3]. Postoperative pain is representative of iatrogenic, acute, and moderate to severe pain in inpatients. Thus, all surgical patients have the right to appropriate pain services, and anesthesiologists bear an important responsibility for such management. However, postoperative pain management is still challenging because of substantial inter-individual variations of pain thresholds, as well as effectiveness of analgesics [4].

In order to increase patient satisfaction with postoperative pain management, multimodal approaches are currently thought to be the most effective [5]. The goal of postoperative pain management is to obtain sufficient pain relief with minimum side effects, regardless of the dosage or type of analgesics. However, appropriate analgesic levels to obtain satisfaction among patients have not been fully clarified.

Pain has been defined as an unpleasant sensory or emotional experience, and thus, self-report is considered to be the most valid measure of pain intensity [6]. In this respect, one plausible hypothesis is that pain management based on each patient’s pain sensitivity may increase their satisfaction levels, e.g., the patient’s satisfaction levels would be high if the level of actual postoperative pain is lower than the acceptable pain they reported preoperatively. If correct, inquiring for the acceptable pain intensity preoperatively would be useful information for setting the goal for postoperative pain management. Therefore, the present study examined whether the differences between estimated acceptable and actual postoperative pain levels may be correlated to the patients’ satisfaction with their postoperative pain management.

### Methods

The study protocol was approved by the ethics committee of Kochi Medical School. The included subjects were scheduled to undergo benign elective surgery under general anesthesia: ASA classes 1–2, age 18–65 years, and those who have the capacity to understand the content of the questionnaire. Participants were excluded if they had previous history of surgery, diagnosed chronic pain, or currently uncontrolled pain. There were no other restrictions with regard to perioperative management, including type of analgesic intervention. The subjects of this study were limited to the patient undergoing benign elective surgery to minimize the possible confounding influence regarding psychological status associated with malignant conditions.

One day before surgery, the patients were asked to estimate their acceptable levels of postoperative pain using a numerical rating scale (NRS) from 0 to 10, where 0 represents no pain and 10 the worst pain imaginable. Postoperative pain controls were conducted by anesthesiologists based on the standard manual of Kochi Medical School Hospital, e.g., non-steroidal anti-inflammatory drugs, intravenous patient-controlled analgesia with fentanyl, or epidural analgesia with local anesthetics and/or fentanyl, according to the type of surgery. One day after surgery, the patients were again asked to rate the average postoperative pain intensity (NRS 0–10) that they had experienced, as well as their overall satisfaction with the provided pain treatment (NRS 0–15; 0 = very unsatisfied, 15 = very satisfied) [7]. The difference between an individual’s estimated acceptable and actual NRS value was calculated by subtracting the postoperative actual NRS from the preoperative estimated acceptable NRS.

#### Statistical analysis

The results of non-parametric data are presented as medians and interquartile ranges. Sample size calculation based on the results obtained from a previous study [8] indicated that a sample size of 87 would have 90% power to detect a significant correlation between pain intensity score and patient satisfaction with overall pain management (*α* error of 0.05). With a 10% allowance for missing data, a sample size of 96 was obtained for this study. Correlation coefficients and *p* values were determined by using the Spearman rank correlation test, and *p* < 0.05 was considered statistically significant. Data were analyzed using the statistical software SPSS (version 11; SPSS Inc., Chicago, IL).

### Results

**Fig. 1:**
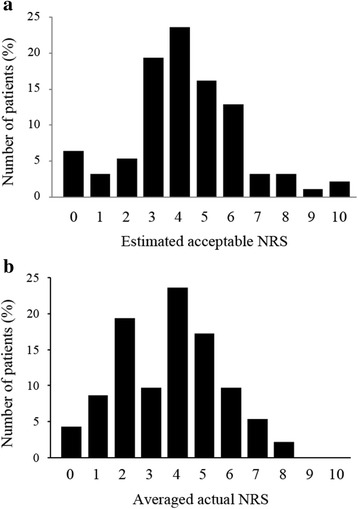
Numeric rating scale (NRS) scores of postoperative pain and patient satisfaction. **a** Distribution of estimated acceptable postoperative pain intensity (NRS 0–10) before surgery. **b** Distribution of actual average postoperative pain intensity (NRS 0–10) after surgery

**Fig. 2:**
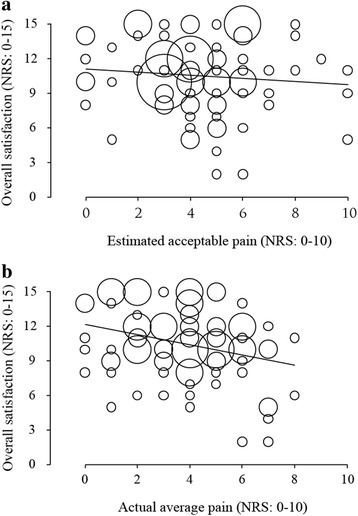
Bubble plots showing the correlation between overall patient satisfaction for postoperative management and estimated acceptable pain intensity (**a**) or actual pain intensity (**b**). The degree of satisfaction and pain intensity were measured by numerical rating scale (NRS) 0–15 and 0–10, respectively. The *lines* represent the regression line. The *circles* are sized according to the number of the same values on the graph

**Fig. 3:**
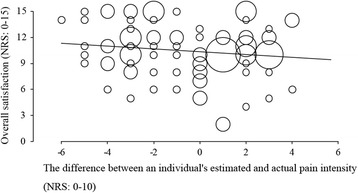
Bubble plots showing the correlation between overall patient satisfaction for postoperative management and the difference between an individual’s estimated acceptable and actual NRS. The degree of satisfaction and pain intensity were measured by numerical rating scale (NRS) 0–15 and 0–10, respectively. The *line* represents the regression line. The *circles* are sized according to the number of the same values on the graph

In this study, among the 96 patients eligible to participate, complete data could be obtained from 93 postoperative patients (97% response rate) and their demographic characteristics are shown in Table 1. The median estimated acceptable and actual NRS values of postoperative pain were 4.0 (3.0–5.0) and 4.0 (2.0–5.0), respectively (Fig. 1a, b). There was no correlation between the degree of patient satisfaction and preoperative estimated acceptable pain intensity (Fig. 2a; Spearman *r* = 0.126, *p* = 0.227). On the other hand, as expected, there was a significant negative correlation between the degree of patient satisfaction and postoperative actual pain intensity (Fig. 2b; Spearman *r* = −0.291, *p* = 0.005). Thirty-four (36.6%) patients rated their actual NRS greater than estimated acceptable NRS, 43 (46.2%) patients rated their actual NRS less than estimated acceptable NRS, and 16 (17.2%) patients rated their actual NRS equal to estimated acceptable NRS. The degree of patient satisfaction was not significantly correlated with the magnitude of difference between preoperative estimated acceptable NRS and postoperative actual NRS (Fig. 3; Spearman *r* = −0.136, *p* = 0.194).

**Table 1:** Clinical and demographic characteristics of the study population (*n* = 93)

Characteristics	Study population
Gender	
Male, *n* (%)	38 (40.9)
Female, *n* (%)	55 (59.1)
Age	
Median (IQR)	48 (37–58)
Type of surgery, *n* (%)	
General surgery	16 (17.2)
Laparoscopic cholecystectomy	10 (10.8)
Hernia repair	6 (6.5)
Orthopedics	32 (34.4)
Spine surgery	12 (12.9)
Total hip arthroplasty	9 (9.7)
Total knee arthroplasty	7 (7.5)
Arthroscopic surgery	4 (4.3)
Gynecology	29 (31.2)
Laparoscopic adnexectomy	15 (16.1)
Laparoscopic hysterectomy	14 (15.1)
Otorhinolaryngology	16 (17.2)
Endoscopic sinus surgery	9 (9.7)
Tympanoplasty	7 (7.5)

### Discussion

Successful pain management may be associated with an increase in patient satisfaction [4]. However, the underlying mechanisms of postoperative pain is complex and affected by not only surgical invasion but also patient characteristics, perioperative pain, anxiety, fear, and patient-medical professional relationships [4, 9, 10]. Furthermore, the pain threshold and sensitivity to analgesics vary markedly among individuals [9, 10]. These factors made it difficult to estimate the severity of postoperative pain in each case.

Similar to our findings, Gerbershagen and colleagues [7] previously reported that they found the correlation between the experienced pain intensity and the patient’s satisfaction. In the present study, our data also demonstrated that the acceptable postoperative pain levels estimated by the preoperative patients themselves varied markedly (Fig. 1a). Interestingly, the median NRS of estimated acceptable pain of our patients was similar with the median value to the estimated tolerable pain levels reported by Gerbershagen and colleagues. However, our study further showed that differences between acceptable pain preoperatively estimated and actually experienced showed no influence in their satisfaction levels (Fig. 3). These findings suggest that it may have been difficult for preoperative patients who had never undergone surgery to imagine postoperative pain and its management accurately. Thus, postoperative pain management based on preoperative patients’ desires may fail to increase their satisfaction. In addition, we cannot rule out the possibility that analgesic regimens based on preoperative patients’ expected acceptable pain levels may lead to excessive analgesic administration and related side effects, which further decrease their satisfaction.

It is now well recognized that comprehensive approaches with multidisciplinary perioperative care teams including anesthesiologists, surgeons, pharmacists, and nurses could improve postoperative pain management [5]. Additionally, adequate preoperative patient understanding for postoperative pain management may increase the quality of postoperative pain management and promote recovery [11, 12]. Moreover, the latest evidence-based guideline for management of postoperative pain [13] highlights the importance of the preoperative patient education. Currently, in Japan, these services are not provided or are not good enough. Therefore, it is imperative to establish preoperative educational programs, e.g., taking medications correctly, managing side effects, non-pharmacologic techniques to reduce pain, and the importance of reporting poorly controlled pain.

There are some limitations in our work which should be addressed. First, patients’ satisfaction was measured by NRS, rather than the commonly applied dichotomous method. However, this allowed us to evaluate the correlation between pain intensity and the degree of satisfaction. Second, in order to investigate the surgical-type dependent impacts, it would be better to ask how much pain a patient expected for the scheduled surgery, rather than the estimated acceptable pain level. Third, in this study, we did not include the elderly population to avoid the potential confounding factor of cognitive impairment. Therefore, further research works including surgery-type and age-specific effects should be conducted.

In conclusion, inquiring about a preoperative patient’s estimated acceptable pain level may not help anesthesiologists set the patient’s goal of pain management to improve their satisfaction.

## Abbreviations

NRS: Numerical rating scale
